# Macular Holes: Main Clinical Presentations, Diagnosis, and Therapies

**DOI:** 10.1155/2022/2270861

**Published:** 2022-04-11

**Authors:** Elias Premi, Simone Donati, Lorenzo Azzi, Giovanni Porta, Cristian Metrangolo, Liviana Fontanel, Francesco Morescalchi, Claudio Azzolini

**Affiliations:** ^1^SC Oculistica, Ospedale di Circolo e Fondazione Macchi, ASST Settelaghi, Varese, Italy; ^2^Department of Biotechnology and Life Sciences, PhD Program, University of Insubria, Varese, Italy; ^3^Department of Medicine and Surgery, University of Insubria, Varese, Italy; ^4^Eye Clinic, Department of Neurological and Vision Sciences, University of Brescia, Brescia, Italy

## Abstract

Macular holes are a spectrum of retinal diseases that comprehends full-thickness macular holes (FTMHs), refractory/recurrent macular holes, lamellar macular holes (LMHs), myopic macular holes (MMHs), traumatic macular holes, and macular holes secondary to other retinal pathologies or injuries. There are various classifications of the subtypes of macular hole, and only in recent times researchers defined a common nomenclature, especially thanks to the evolution in retinal imaging, offered by new instruments like the swept-source OCT. The proposed therapies for macular holes are different and range from a “wait-and-see” approach to the vitrectomy, with different results in each subtype of macular hole. This narrative review has the purpose to investigate the available evidence in literature to give a summary of the knowledge about these retinal pathologies.

## 1. Introduction

Macular holes are a wide spectrum of retinal diseases that comprehends different subtypes, each of them with its defined pathogenesis, morphology, and specific therapeutic option. Clinical presentations could also be very different and range from asymptomatic to visual impairment associated with strong metamorphopsia. Idiopathic macular hole (IMH), also known as full-thickness macular hole (FTMH), was first classified in four stages and discussed by Gass on fundus observation in 1988 [1]. Lamellar macular hole (LMH) is a different pathology, described for the first time by Gass in 1975 [2] and for a long time classified into two different subtypes known as tractional lamellar macular hole (tLMH) and degenerative lamellar macular hole (dLMH) [3]. Myopic macular hole (MMH) is a particular subtype that belongs to a wider group of retinal diseases called myopic tractional maculopathy (MTM) and shows some peculiar clinical aspects [4]. Traumatic macular hole is secondary to ocular trauma and shows specific features and clinical evolution. Secondary macular holes present different etiologies and have to be discussed separately. Actually, the continuous improvement in diagnostic instruments, such as optical coherence tomography (OCT), has led to a better understanding of these diseases, even if some aspects still have to be clarified [5]. “Refractory macular hole” is a term that refers to the FTMH that do not close after the surgery for primary FTMH or reopen after a complete surgical closure. It is a challenging disease for surgeons because the surgical techniques change due to the results of first surgery. Recently, new, various, and innovative techniques have been proposed to treat refractory macular holes.

The purpose of this review is to summarize the most recent available evidence in literature about these pathologies and in particular their pathogenesis, classification, and therapeutic options.

## 2. Methods

A literature search was conducted in the PubMed database (https://pubmed.ncbi.nlm.nih.gov/) using keywords “macular hole,” “full thickness macular hole,” “lamellar macular hole,” “myopic macular hole,” and in addition, for every subtype, a literature search has been performed combining previous keywords with “pathogenesis” and “surgery OR treatment.” Only peer-reviewed articles were considered.

## 3. Main Text

### 3.1. Full-Thickness Macular Hole (FTMH)

**Figure 1:**
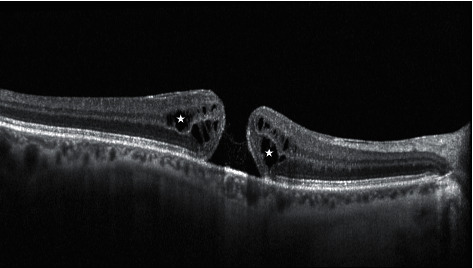
SD-OCT scan of a full-thickness macular hole (FTMH). In this scan, the white stars highlight the presence of intraretinal cysts of edema. In this image, there is a complete absence of the Muller cone complex and the vitreous is totally detached and not visible.

FTMH is defined as a foveal defect that interests all retinal layers, from internal limiting membrane (ILM) to photoreceptor layer (ellipsoid zone or IS/OS junction), and it may be associated with a severe vision impairment [1] (Figure 1). Idiopathic macular hole is nowadays a term that is outdated in scientific literature due to a better comprehension of the widely accepted pathogenesis of the disease: a pathological anteroposterior traction of the vitreous cortex on the fovea and perifoveal area [6].

Anteroposterior traction does not represent the only mechanism involved in this pathology. Other vectorial forces have a role in the progression of the FTMH. Retinal tangential tractional forces seem to play an important role in the enlargement of the hole and in the elevation of its edges: these vectors are determined by ILM stiffening that occurs after the separation of the posterior vitreous cortex from the foveal retina. Another important aspect to be considered in edges elevation and cysts formation (Figure 1) is the hydration mechanism of retinal layers after the exposition to the vitreous fluid [7], associated with a loss of function of Muller cells ripped from the fovea [8]. Muller cells are fundamental actors in the retina, as they guarantee the integrity of the anatomy and a normal homeostasis of the tissue: in the foveal region, the particular structure of Muller cells on a cone shape (macular cell cones, MCCs) occupies one-third of the entire retinal thickness [9] and weakens the resistance of the retina to anteroposterior forces, explaining the origin of FTMH [10]. Recently, dynamic forces have also been identified to play a role in the FTMH pathogenesis: the continuous movements of the eye during the day can cause a rotational swing of the vitreous and the generation of fluid currents that appear to be more intense in patients who developed an FTMH after posterior vitreous separation [11].

Patients with FTMH show some alterations on the inflammatory profile of the aqueous humor: a recent study showed that IL-1r*α*, IL-4, IL-13, IL-15, IL-1*α*, IL-2R*α*, IL-3, IL-12p40, IL-18, hepatocyte growth factor (HGF), GM-CSF, IFN-*γ*, MIP-1*α*, PDGF-bb, LIF, M-CSF, *β*-NGF, SDF-1*α*, TNF-*β*, and TRAIL*γ* were significantly upregulated in the experimental group, while IL-5, eotaxin, IP-10, TNF-*α*, GRO*α*, IL-7, and MIF were significantly downregulated after the formation of FTMH [12]. The role of these cytokines is still unclear, but it is a promising step to better understand the inflammatory pathogenesis of FTMH.

Considering the classification, the most widely used and accepted is the OCT-based staging proposed in 2013 by the International Vitreomacular Traction Study (IVTS) group [13] that updated the previous funduscopic classification by Gass [1]. In this classification, FTMHs are associated (early stage) or not (end stage) with a vitreomacular traction (VMT) derived from a vitreomacular adhesion (VMA). Furthermore, this classification divides FTMHs into three subgroups based on the horizontally measured linear width at the narrowest point of the hole: small (≤250 *μ*m), medium (>250 *μ*m and ≤400 *μ*m), and large (>400 *μ*m). Recently, some authors discussed the possibility to define a large FTMH greater than 650 *μ*m, in particular after clinical observations of a high successful rate of macular hole closure after standard surgery in patients presenting with FTMH of linear width less than 650 *μ*m [14].

Nowadays, it lacks a clear and shared consensus on surgical technique to manage ILM peeling and on patient positioning after the surgery to guarantee an effective FTMH closure. Small-gauge pars plana vitrectomy with gas or air tamponade is widely accepted as the gold standard technique [15], showing a high percentage of FTMH closure [16].

Focusing on the ILM peeling technique, several studies have been recently conducted to evaluate the extension of the circular area of peeling. In these studies, it has been observed that a larger diameter of the peeling is associated with a higher rate of FTMH closure, but it does not correlate to a better best-corrected visual acuity (BCVA) after surgery. In particular, four disc diameters (DD) peeling versus 2 DD peeling showed a better anatomical result [17, 18] and 2DD peeling showed a better anatomical result versus 1 DD peeling [19]: these results were significant in particular in large FTMHs. Today, ILM peeling is considered mandatory in almost all FTMH surgeries, considering a higher rate of FTMH closure compared with the simple removal of the posterior vitreous cortex [16, 20]. The rationale of ILM peeling is to remove all wisps of vitreous cortex and to reduce the scaffold for cell proliferation into a contractile epiretinal membrane (ERM) that could fail the surgery and induce FTMH re-opening.

In the past years, the studies on different types of ILM peeling have been performed with several new techniques to increase the FTMH closure rate, even in many cases it does not correlate to a better postoperative BCVA [21].

The foveal sparing technique is performed by peeling the ILM on a circular way with respect to the ILM surrounding the FTMH: this approach aims to preserve Muller cells still intact around the macular hole. Some studies showed a better improvement of the postoperative BCVA [22] and retinal sensitivity [23] compared with a complete ILM peeling.

ILM flap techniques represent a different way to manage ILM peeling in macular hole surgery, and they showed great results in anatomical restoration of foveal profile, especially in larger and recurrent FTMHs [24]. A large number of ILM flap creation and management techniques have been proposed in the past years. In flap techniques, the ILM behaves as a scaffold and promotes, together with Muller cell remnants, a gliosis phenomenon around the macular hole, inducing the migration of photoreceptors in the correct position [25]. One of the most studied procedures is the inverted flap technique, in which the ILM is centripetally peeled around the fovea, but it remains adherent to the edges of the macular hole [26]. This technique presents some variants in the management of the flap, which could be plugged inside the macular hole to fill the empty space or laid on the top of the macular hole. These two variants imply different patterns of FTMH closure: by covering the macular hole, it gives a precious aid to control intraretinal diffusion of fluid and to restore retinal layers, but in some patients a delay or a lack in restoration of outer layers is observed [27]; on the contrary, by plugging the ILM inside the hole, it helps all retinal layers to restore, acting as a filler, glue, and finally a scaffold [28]. Few data are available in literature about differences in functional outcomes between these techniques: by covering the hole with the ILM flap, it seems to be associated with better functional outcomes [29], even if there are no evidence of these results in very large macular holes; by plugging the ILM in the hole, it appears to guarantee a better rate of FTMH closure [28, 29]. Temporal inverted flap technique is the last modified version of this kind of surgical strategy that aims to preserve the surgical damage caused by ILM peeling on the nerve fiber layer in the area between the optic disk and the macula: this technique showed same results in the FTMH closure rate as the classic inverted flap technique as shown before [30].

Free-flap technique presents the same rationale of other flap techniques but is characterized by the use of an ILM flap picked up outside the macular region without any contact with its edges. This technique is very useful in cases of refractory FTMH, when the ILM is no more available around the hole due to previous peeling; this approach does not represent the first choice when the ILM is still available around the FTMH because in this case inverted flap technique guarantees a safer positioning of the flap, reducing the risk of losing the flap during surgical maneuvers [25].

Recently, other scaffolds for glial cell proliferation have been proposed to increase the percentage of surgical success in FTMHs, especially in large FTMHs. Lens capsule flap is an autologous membrane that could be obtained from the anterior or posterior capsule of the lens and dropped inside the FTMH to offer a scaffold to glial cells to proliferate and promote the closure of the macular hole as the ILM does: a recent study showed a higher FTMH closure rate when the anterior lens capsule was used than posterior lens capsule [31]. Another organic tissue that has been proposed is human amniotic membrane (hAM), generally used in corneal surgery. Recent evidence showed that it could be used as a plug to be inserted inside the FTMH. The amniotic membrane plug has to be correctly processed and positioned inside the hole to guarantee a good stability: the plug needs to have a proper dimension to be positioned under the retina, and the amniotic chorion layer must be in touch with the retinal pigmented epithelium (RPE). This technique showed a great successful closure rate and a BCVA improvement in large FTMHs [32, 33]. Postoperative OCT evaluation after positioning of amniotic membrane plug showed the progression of overlying retina and the appearance of anatomical retinal layers [33].

Experimental research also evaluated liquid plugs. The use of autologous whole blood (AWB), autologous serum (AS), autologous platelet-rich plasma (aPRP), and thrombin has been described to promote macular hole closure [34]. Recent evidence showed that the use of aPRP significantly improves the anatomical and functional results of FTMH treatment, probably enhancing glial proliferation, which ensures FTMH closure [35].

FTMH surgical treatment results also depend on postsurgical patient posturing. Intraocular gas tamponade needs for a correct postsurgical patient positioning for the right time to ensure best results, avoiding unnecessary long-time face-down posturing that could be uncomfortable for the patients. Recent evidence shows that short-acting gas tamponade reaches same FTMH closure rates compared with long-lasting gas tamponade [36–38]. Worldwide, prone position is the most suggested behavior for patients who underwent macular surgery for FTMH [39]. A recent randomized controlled trial showed not significant differences in FTMH closure rates between patients who keep face-down positioning (FDP) and who keep a non-face-down positioning (nFDP) from the day after surgery, but it is to be noted that in this RCT only few patients presented a macular hole larger than >400 nm [40]. This report appears to be in accordance with a recent meta-analysis that showed no differences in FTMH closure rates, considering different postsurgical positioning in the subgroup with macular hole smaller than 400 nm; however, they reported a significant difference in the FTMH closure rate if we consider FTMH of any dimension or FTMH larger than 400 nm in patients who keep FDP [41, 42].

An interesting new approach in managing patient positioning after the surgery is the OCT-guided posturing. OCT in the early postoperative period is reported to be a useful tool to determine whether an MH is closed even in gas-filled eyes. Longer wavelength OCT scans, as obtained by swept-source OCT technology (SS-OCT), can reach a higher penetration through the opaque media, offering a good image of the macula even if the eye is fulfilled with gas tamponade. The SS-OCT was reported to be able to determine the MH status in gas-filled eyes as early as 2 hours after surgery [43]. Using this technology, the duration of the face-down positioning after MH surgery could be greatly reduced, avoiding unnecessary posturing [44].

The application of OCT control for monitoring FTMH healing after surgery could be useful to reach complete FTMH closure, minimizing discomfort for the patients. OCT is also useful to evaluate the complete healing process of the lesion during time, especially to evaluate the presence of the outer retinal layers [45].

FTMHs are still an interesting topic of discussion and research, considering different types of surgical approaches that have been proposed and still evolving and the possibility to introduce postoperative monitoring with OCT imaging.

### 3.2. Refractory FTMH

The surgical techniques showed in the previous section ensure a high rate of FTMH closure, but in a percentage up to 10% the FTMH may not close and so be defined as a refractory FTMH [46]. Persistence of FTMH have some identified risk factors, such as a size greater than 500 *μ*m, long-lasting FTMH more than 6 months, high myopia, incomplete ILM peeling or incomplete filling of gas tamponade, inability of the patient to keep the postoperative posturing, traumatic etiology, atrophic hole configuration (flat edges), previous uveitis, and concomitant age-related macular degeneration [47].

An FTMH could be defined refractory also when it recurs after a complete primary closure [48]. Identified risk factors for recurrent FTMH are axial length greater than 26 mm, cystoid macular edema, and presence of atrophic age-related macular degeneration [48].

Various treatments of refractory FTMHs are available and are focused on the same rationale of treatment for primary FTMH closure, but the frequent lack of ILM already peeled leads to the choice of different solutions, in order to remove tangential forces, increase retinal mobility, and plug the hole to promote gliosis [49].

As presented in the previous section, the ILM represents one of the main causes of tangential traction on the retina and if not sufficiently removed could cause recurrence of macular hole. A new intrasurgical ILM staining and removal by enlarging its area could represent a therapeutic possibility in order to close the refractory FTMH [50]. Recent studies and meta-analyses show that good anatomical results and gain in functional outcomes could be achieved by reoperating the refractory FTMH using this technique, which could also be improved with the free flap of ILM inside the hole [48, 49].

As in the primary treatment of FTMH, the ILM free-flap management could be challenging; in particular, to insert it exactly inside the hole and to keep it in position, especially during fluid-air exchange procedures. The use of perfluorocarbon liquid (PFCL) can help maintain the flap in position on the macular hole during the procedure and also to facilitate the ILM peeling itself [51, 52]. Another surgical option is to maintain the free flap on the hole using ophthalmic viscosurgical devices (OVDs) [53]. Eye gas filling and face-down positioning are mandatory to improve the rate of refractory macular hole closure when using these techniques.

Other techniques that could be chosen to plug macular holes than the free-flap ILM are the same showed before in the section of primary FTMH: lens capsule, the hAM, and aPRP [31–33, 35].

Increasing retinal mobility is another choice that could be applied to induce closure of recurrent FTMHs. Relaxing retinotomies are used to increase the mobility of the retina and to reduce the traction of the macular hole surrounding the retina. The temporal retina is cut with scissors on an arciform shape and displaced nasally to close the macular hole. After these surgical procedures, maneuvers, the patient must keep face-down positioning with a gas full-filled eye for 7 days [54]. Even if the closure rate evidenced in literature is high, the BCVA does not show a concomitant significant improvement and a paracentral scotoma may develop. Several authors suggest that these relaxing retinotomies may not be considered as the first choice in recurrent FTMHs, considering other available surgical procedures [55]. Again, to allow retinal mobility, several authors described the induction of an iatrogenic, localized retinal detachment surrounding the refractory FTMH by the subretinal injection of balanced saline solution (BSS) through small caliper cannulas (41G). The injected BSS forms little blebs surrounding the refractory FTMH, which subsequently need to merge with the macular hole [56].

Recent literature reports showed a high rate of refractory FTMH closure and a significant improvement in BCVA [56].

In conclusion, recurrent FTMHs still represent a challenging form of macular holes to be treated by vitreoretinal surgeons. Nowadays, many therapeutic options are available, allowing to reach a good anatomical closure rate, when correctly applied.

### 3.3. Lamellar Macular Hole (LMH)

Lamellar macular hole (LMH) is a vitreoretinal disorder that affects the macular region, and it is characterized by defined anatomical features: an irregular foveal contour, a break in the inner fovea, dehiscence of the inner foveal retina from the outer retina, and the absence of a full-thickness foveal defect with intact foveal photoreceptors [3]. Recent meta-analysis showed a prevalence of LMH in the general population ranging from 1.1 to 3.6%, with no significant correlation with gender and age [57]. LMHs show slight symptoms onset and in early stages are asymptomatic in many cases. When it occurs, symptoms are decreased BCVA, metamorphopsia, and slight central scotoma. The LMH is generally idiopathic but could be secondary to retinal diseases [58–61], choroiditis, ocular trauma [62], systemic diseases [63], or iatrogenic [64].

**Figure 2:**
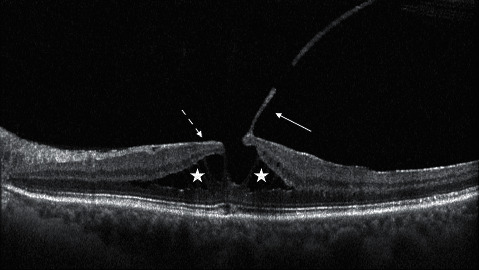
An epiretinal membrane associated with foveoschisis in its SD-OCT appearance. The white stars show the foveoschisis. The arrows show the presence of an adherent epiretinal membrane (dashed line) and vitreous cortex (continue line).

**Figure 3:**
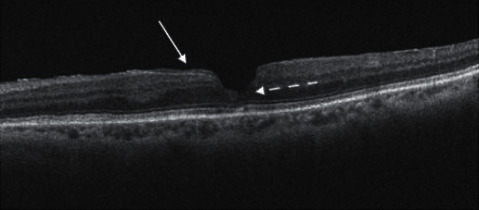
SD-OCT scan of a lamellar macular hole. In this scan, the white arrow shows the presence of the iso-reflective epiretinal proliferation. The dashed white arrow highlights the irregular foveal contour and the initial disruption of outer retinal layers, especially external limiting membrane and ellipsoid zone.

Nowadays, a recent consensus on OCT definition of LMH established a new classification that divided LMHs in three subgroups: LMH, epiretinal membrane (ERM) associated with foveoschisis, and macular pseudo-hole (MPH). Each entity is defined by mandatory and optional criteria. The LMH definition is based on three mandatory criteria (presence of irregular foveal contour, presence of a foveal cavity with undermined edges, and apparent loss of foveal tissue) and three optional anatomical features (presence of epiretinal proliferation, presence of a central foveal bump, and disruption of the ellipsoid zone) [65] (Figure 2). The ERM foveoschisis definition is based on two mandatory criteria (presence of ERM and retinal schisis at the level of Henle's fiber layer) and on three optional anatomical features (presence of micro cystoid spaces in the inner nuclear layer (INL), an increase of retinal thickness, and presence of retinal wrinkling) [65] (Figure 3). The MPH definition is based on three mandatory criteria (presence of a foveal sparing ERM, the presence of a steepened foveal profile, and an increased central retinal thickness) and two optional anatomical features (presence of micro cystoid spaces in the INL and a normal retinal thickness) [65].

This new classification has been created, thanks to a better understanding of the pathogenesis of this disease. Recent evidence shows that the ERM and epiretinal proliferation present deep anatomical and pathological differences in particular on OCT evaluation. These two types of epiretinal membranes are identified in almost all LMHs [66, 67]. ERM foveoschisis is associated with an epiretinal membrane similar to a macular pucker, characterized by bright hyper-reflective aspect at OCT scans. Moreover, this ERM shows an immunohistochemical pattern featured by the strong presence of *α*-smooth muscle actin (*α*-SMA) [66], explaining the contractile behavior of this kind of membrane. On the other side, epiretinal proliferation appears as an iso-reflective, thick membrane on OCT scans and presents a different immunohistochemical pattern, characterized by the presence of compact fibrous long-spacing collagen, as a product of degradation of normal collagen fibrils. Both kinds of membrane show some common immunohistochemical profiles, such as CD45, CD64, and glial fibrillary acidic protein: these biomarkers are typically expressed by hyalocytes and glial cells, underlying the role of these cells in the pathogenesis of this macular disease [66]. The differences between these two clinical features gain more and more evidence if we consider their progression and impact on visual function, that is, more severe in LMH with epiretinal proliferation, probably due to deeper alterations in outer retinal layers [68], in particular to the ELM [69].

As presented before, OCT plays an important role in the evaluation of LMH structure and morphology. Recent studies have shown interesting results using short-wavelength fundus autofluorescence (SW-FAF) [70] and microperimetry to assess retinal sensitivity (RS) [68, 70]. Larger LMHs show hyper-FAF area combined to lower preoperative RS. After surgery, the hyper-FAF area decreased, and RS increased. The reduction of FAF to physiological features is associated with higher RS and a whole functional improvement [70]. These further evaluations will help to better evaluate surgical indications for LMH.

LMH surgical treatment and particularly its timing are still controversial. No agreement has been reached about the management of patients affected by LMH: observation and follow-up or surgery to peel ERM and ILM. Surgical recommendations seem to be more frequent in European countries than in the USA. Several studies show that the natural history of LMHs is characterized by an overall stability and a little progression of signs and symptoms [71]; only few cases progress into an FTMH [72] or show a decrease in BCVA. Today, surgical treatment is proposed only for patients with a documented progression of the lesion over time and a worsening of symptoms. The widespread and approved technique is pars plana vitrectomy with ERM and ILM peeling; some authors also suggest to use gas tamponade to promote the closure of LMH [73, 74], but some others demonstrated that the vitrectomy with ERM and ILM peeling alone is sufficient to stimulate the restoration of foveal profile and integrity [75, 76]. Most recently, available data about surgical outcomes on LMHs show a slight difference between ERM foveoschisis and epiretinal proliferation groups. Both groups showed an increase in BCVA after surgical treatment [77], but the ERM foveoschisis group demonstrated a significant increase in BCVA when compared with the LMH with epiretinal proliferation group [78]. Furthermore, patients presenting LMH with epiretinal proliferation showed an increased risk to develop an FTMH after surgical procedure [77], probably due to damage to Muller cells during surgical removal of epiretinal proliferation, strongly adherent to the retina [66]. This controversial evidence suggests to always evaluate the surgical risk-to-benefit ratio in every LMH patient [57].

Recently, to reduce the risk of postoperative FTMH, some surgical options have been proposed. Shiraga et al. recommended that the epiretinal proliferation tissue containing yellowish pigment over the LMH should not be removed to elicit normalization of the contour of the fovea [79]. Moreover, a new technique of double inverted ERM and ILM flap was proposed to treat LMH. This surgical technique is based on the construction of a double inverted ERM and ILM flap around the edges of the LMH, following the concept of the inverted flap technique proposed by Michalewska et al. for the treatment of large FTMHs. The surgical rationale is to release the tangential traction on the LMH without complete removal of ERM and ILM from the edges of LMH, and the creation of 3 or 4 flaps to take the advantages of the properties of ERM and ILM flaps to induce cell reproliferation and restoration of the foveal architecture [70].

### 3.4. Myopic Macular Hole (MMH)

**Figure 4:**
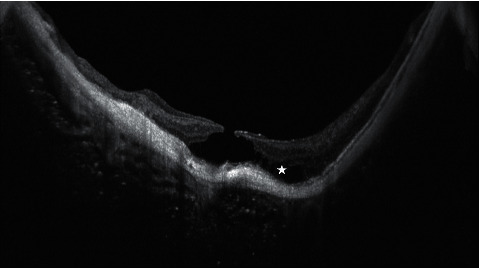
Full-thickness myopic macular hole. In this SD-OCT scan, the myopic profile and staphyloma of the posterior pole are evident. The sclera and the choroid are very thin. The white star highlights the initial stage of a myopic foveal schisis.

Myopic macular holes (MMHs) are clinical entities that belong to a wider group of retinal diseases defined as myopic traction maculopathy (MTM) [80]. MTM includes the following alterations: foveoschisis/maculoschisis/retinoschisis (FS/MS/RS), retinal/foveal detachment (RD/FD), lamellar macular holes (LMHs), and full-thickness macular holes (FTMHs) with MHRD or without RD [4]. Generally, myopic LMHs and FTMHs present a similar clinical aspect with the interruption of retinal layers as nonmyopic macular holes (Figure 4). In MTM, however, the tangential and centripetal epiretinal vectorial forces are slightly different and must be considered, as also the different pathological behavior and progression [81].

Myopic FTMH represents a severe complication of myopic traction maculopathy. Two different types of vectorial forces act on the retina: preretinal forces and subretinal forces. Preretinal forces are centrifugal but also tangential and are determined by epiretinal membranes, vitreomacular traction, and incomplete posterior vitreous detachment [82]. Moreover, the retinal blood vessels network contributes to the stiffness of the retina. Subretinal forces are caused by the changes of the sclera in the myopic eye and by the staphyloma, which influence the radius of curvature of the eye wall [83]. Patients with myopic FTMH could develop a macular retinal detachment, which represents the most severe complication. Some authors identified the association of myopic FTMH with the presence of foveoschisis [84] as a risk factor for the progression to the retinal detachment. All surgical treatments for myopic FTMH are addressed to reduce the vectorial forces that act on the retina. First, an “ab interno” approach requires vitrectomy and the removal of all tangential tractions, in particular, remnants of vitreous cortex, epiretinal membranes, and ILM to reduce the stiffness of the retina; second, the “ab externo” technique prevents MH formation and is focused to limit subretinal forces caused by the modifications of the sclera by means of macular buckling that reduces the curvature radius of the posterior pole and the progression of the staphyloma [81].

Myopic LMHs are quite similar to nonmyopic lamellar macular holes. They are divided into two subgroups according to the presence of a typical or atypical ERM. This distinction is not only morphological but seems to be associated with a different behavior of these lesions. Myopic LMH associated with atypical ERM shows a worse visual prognosis and the risk to evolve into a myopic FTMH [85].

### 3.5. Secondary or Atypical Macular Hole

Secondary macular holes differ in etiopathogenesis and clinical features and must be discussed individually.

Macular telangiectasia (MacTel) is a retinal disease of unknown etiology, always bilateral and acquired. Recent studies observed alteration in the Muller cell cone that guarantees the structure of foveola. OCT scans in MacTel show the presence of retinal tissue cavitations in the foveal area with ILM sparing until last stages [86]. The fusion of several cavitations could lead to a damage of ILM with the dislocation of photoreceptors and the development of an FTMH, probably associated with concomitant VMT [60].

Retinal artery macro aneurism is a dilatation of a retinal vessel that origins in any part of the retina, usually at the posterior pole. The vessel wall dilatation is due to systemic conditions such as hypertension or diabetes 75, [87, 88]. Alterations of the blood flow could cause the macro aneurism rupture with bleeding in subretinal space or in intraretinal layers. The hemorrhage blood pressure in the subretinal space and the retinal structure degeneration due to the blood degradation could cause FTMH formation [89]. This pathological mechanism is also observed in rare cases of wet AMD patients with a subretinal hemorrhage.

Cystoid macular edema is a complication of several retinal diseases such as diabetic retinopathy, retinal vein occlusion, and posterior uveitis [90]. The common pathological pathway that leads to FTMH formation is the coalescence of intraretinal cysts and the structural modification of intraretinal layers. In some cases, a tangential vectorial force is present, like an epiretinal membrane that acts as a cofactor in the FTMH formation [91].

Ocular surgery may develop an iatrogenic FTMH formation, a well-known complication. Postsurgical FTMH could develop after different types of surgeries; during cataract surgery, if complicated by posterior capsular bag rupture and vitreous loss. Vitreous liquefaction associated with vitreomacular traction and the development of postsurgical inflammatory macular edema could develop a macular hole [92, 93]. FTMH formation could be also a complication of vitreoretinal surgery for retinal detachment, in particular after pneumoretinopexy [94]. The pathological mechanisms are the incomplete posterior hyaloid detachment in the macular region associated with the presence of an epiretinal membrane, cystoid macular edema vitreomacular traction, and more the injection of gas bubbles in nonvitrectomized eyes [94, 95].

At last, laser devices could be the cause of an FTMH, due to the direct damage of the laser beam on the retinal tissue. Many case reports show FTMH formation after laser procedures or accidental exposition. The Nd-Yag laser, used for capsulotomy or vitreolysis procedures, when focused on the retina, causes plasma formation and micro-explosions that could damage the central retina with FTMH formation [96]. Also, handheld lasers could damage the retina if the beam reaches the retinal tissue, in particular, if they are high-powered laser, causing not only a traumatic macular hole but a real photocoagulation and atrophic FTMH [97].

### 3.6. Traumatic Macular Hole

Blunt ocular trauma is a common event and could lead to a traumatic FTMH. Many FTMHs have been observed after ocular trauma, and two different pathological pathways have been proposed to explain the FTMH formation. The first one is the anteroposterior acute traction exerted on the retina by the vitreous during the compression and rebound of the globe due to trauma forces; the second is the tangential elongation of the globe on the equator and the horizontal stretch of the retina [98]. Traumatic macular hole could also result from progressive retinal atrophy due to commotio retinae [99]. The FTMH formation leads to an acute visual impairment suddenly after the trauma, even if some studies report a subacute onset in a few weeks. Clinical features of the FTMH are an oval aspect and the presence of a surrounding subretinal fluid, sometimes associated with Berlin's edema or a subretinal hemorrhage [98]. In these cases, the surgical approach with pars plana vitrectomy and ILM peeling offers good results [100], even if several reports show spontaneous resolution of traumatic FTMHs in a few weeks following the trauma [101].

## 4. Conclusions

Macular hole is a diversified retinal pathology that could affect visual acuity of a patient with a severe prognosis. Even if the progression of macular surgery techniques demonstrated to be able to improve visual acuity in most cases, sometimes macular holes represent a hard surgical challenge and the risk of several recurrences. Moreover, despite the surgical success, patients still experience metamorphopsia or little scotomas that could not be resolved at all, causing frustration for patients and surgeons. Today different solutions are available to treat all subtypes of macular holes, but a global consensus is not yet available on which technique guarantees the best surgical outcomes. As for the treatment options, also the classification still lacks a definite consensus, and this fact affects homogeneous research and meta-analysis to better understand this disease. Recent published classifications on FTMH, LMH, and MMH based on OCT evaluation will be useful to standardize clinical studies. Future randomized clinical trials will better investigate treatment outcomes for a shared classified subtype of macular holes [87].

## Data Availability

No data were used to support this study.
